# Adenocarcinoma With Intestinal and Pancreatobiliary Features Arising From a Sacrococcygeal Teratoma in an Adult Female: A Case Report

**DOI:** 10.1155/crip/5088951

**Published:** 2025-08-10

**Authors:** Yasamin Mirzabeigi, Clara Milikowski

**Affiliations:** ^1^Department of Pathology and Laboratory Medicine, Jackson Memorial Hospital/University of Miami, Miami, Florida, USA; ^2^Department of Pathology and Laboratory Medicine, University of Miami Miller School of Medicine, Miami, Florida, USA

**Keywords:** case report, malignant transformation, sacrococcygeal teratoma (SCT)

## Abstract

Sacrococcygeal teratomas (SCTs) are rare in adults, and malignant transformation within these tumors is exceedingly uncommon. The risk of malignant transformation among adults can vary between 1% and 12% and increases over time with the endopelvic location. Here, we present a case of invasive adenocarcinoma with intestinal and pancreatobiliary features arising from a preexisting SCT in a 64-year-old female. The patient presented with a rapidly enlarging sacral mass and purulent drainage decades after the excision of a congenital lump in the sacral region. Imaging revealed a lytic lesion involving the coccyx and sacrum, accompanied by an ill-defined soft tissue mass. Histological evaluation confirmed adenocarcinoma with dual intestinal and pancreatobiliary differentiation originating from a preexisting SCT with focal involvement of the resection margin. Postoperatively, a multidisciplinary team recommended FOLFIRINOX chemotherapy followed by completion excision surgery. This case contributes to the limited literature on adult SCTs with malignant transformation, highlighting the critical need for timely and comprehensive management. Multidisciplinary evaluation, complete surgical resection, and tailored adjuvant therapy are essential to improving patient outcomes in such rare cases.

## 1. Introduction

Sacrococcygeal teratoma (SCT) is the most common extragonadal teratoma, accounting for 40%–50% of extragonadal Type 1 germ cell tumors. The reported incidence is 3.7–7.1 cases per 100,000 live births and a male-to-female ratio of 1:3 to 4 [1–6]. While SCTs are usually found in the prepubertal population, they are exceedingly rare in adults [7]. SCTs arise from the remnants of the primitive streak and contain layers of pluripotent embryonic germ cells from any of the three primitive cell layers [8].

Based on the anatomical location, SCTs are divided into four categories. Type I sacrococcygeal tumors (46%) are entirely external to the pelvis, while Type II (35%) are primarily external with a minor intrapelvic component. Type III (9%) tumors are predominantly pelvic with a small external portion, and Type IV (10%) are completely contained within the pelvis. Together, Types I and II constitute 80% of SCTs and are most frequently found in neonates, often allowing for early diagnosis and surgical removal. In adults, however, SCTs are more commonly of Type III or IV [9].

SCTs are generally benign, but rare cases of malignant transformation have been reported in the literature. The risk of malignant transformation among adults can vary between 1% and 12% [10] and increases over time with the endopelvic location [11]. Malignancy of any component may occur, although ectodermal squamous cell carcinoma followed by adenocarcinoma are the most common [12]. Herein, we report a case of invasive adenocarcinoma with primarily intestinal and pancreatobiliary features arising from a preexisting SCT in a 64-year-old woman.

## 2. Case Report

A 64-year-old female presented with a history of a congenital lump in the sacral region that was surgically excised at the age of 2. She remained asymptomatic for decades until recently, when she developed a rapidly enlarging mass in the same area, accompanied by purulent drainage. Initially diagnosed as a pilonidal cyst, the mass was treated with multiple courses of antibiotics, but there was no response.

**Figure 1:**
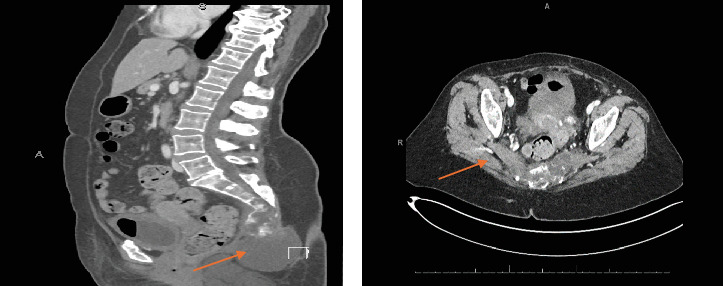
Preoperative CT scan showing an ill-defined soft tissue mass (arrows) in the coccygeal and lower sacral region. (a) Sagittal view. (b) Coronal view.

During further clinical workup, a computed tomography (CT) scan revealed a lytic lesion involving the coccyx and lower sacrum, accompanied by an ill-defined soft tissue mass measuring 4.7 × 5 × 6.3 cm (AP × TR × CC). The mass contained multiple hypodense areas and extended into the presacral space (Figure 1).

The patient eventually underwent surgical resection of the mass.

Grossly, the specimen measured 5.5 × 4.5 × 4.3 cm and consisted of yellow-tan soft tissue partially covered by a 6 × 2 cm gray-tan ellipse of skin. The deep aspect of the specimen was focally disrupted. Serial sectioning revealed an approximately 5 × 4 × 3.5 cm cystic lesion with focal calcifications and septations, filled with red-tan hemorrhagic friable tissue.

**Figure 2:**
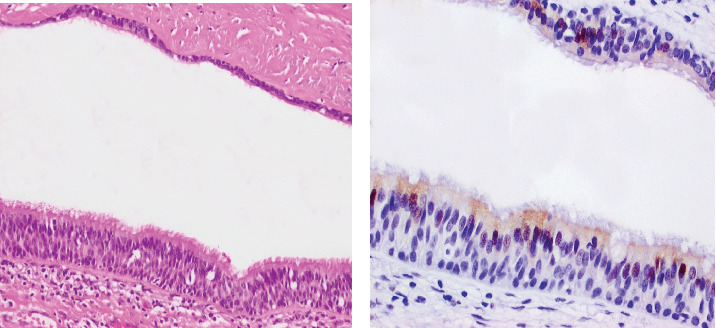
(a) Pseudostratified ciliated columnar respiratory epithelium on H&E stain. (b) Respiratory epithelium highlighted by TTF-1 immunostaining.

**Figure 3:**
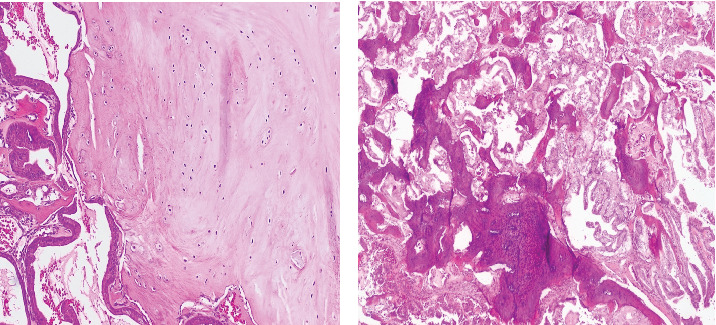
(a) Fibrocartilage and (b) woven bone infiltrated by the adenocarcinoma component.

Histological evaluation revealed a cystic neoplasm composed of foci of benign pseudostratified ciliated columnar respiratory epithelium, which were positive for TTF-1 immunohistochemistry (IHC) staining (Figure 2). Additionally, areas of abundant woven bone, along with hyaline and fibrocartilage formation, supported the diagnosis of preexisting SCT (Figure 3).

The majority of the neoplasm consisted of multilayered, intestinal-type, mucin-producing glands with architectural complexity and cytological atypia, invading the surrounding stroma. On IHC, the glands with intestinal features were positive for CDX-2, faintly positive for CK7, and retained SMAD4 expression.

**Figure 4:**
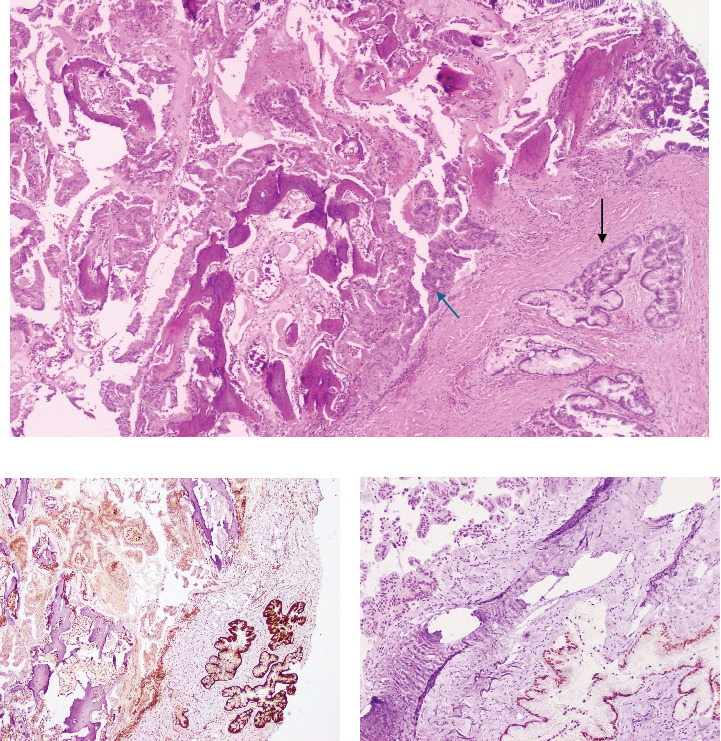
(a) Intestinal-type mucinous glands (black arrow) and pancreatobiliary glands (blue arrow) on H&E stain. (b) CDX2 immunostaining shows positivity in intestinal glands and negativity in pancreatobiliary glands. (c) Loss of nuclear SMAD4 staining in pancreatobiliary glands, with retention in intestinal-type glands.

In contrast, a separate population of infiltrative glands, growing in a haphazard fashion, displayed strong CK7 positivity and loss of SMAD4 expression. These features were more consistent with adenocarcinoma exhibiting pancreatobiliary differentiation (Figure 4).

Although disruption of the specimen precluded precise assessment of all surgical margins, carcinoma was noted to focally involve the inked resection margin.

**Figure 5:**
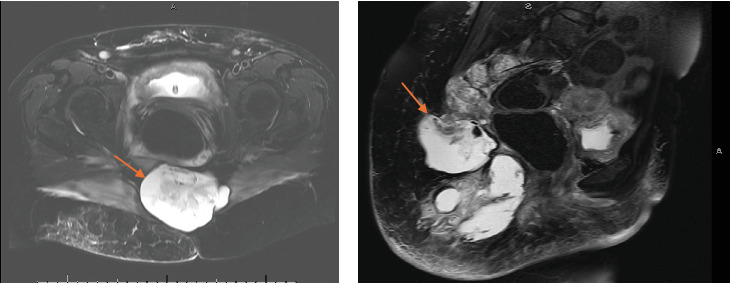
The 6-week postoperative MRI showed a solid cystic mass (arrows) infiltrating the sacrum with solid cranial and cystic caudal components. (a) Sagittal view. (b) Coronal view.

The patient's postoperative course was complicated by sacral abscess formation and persistent growth of the mass. An abdominal and pelvic MRI performed 6 weeks after surgery revealed a large cystic mass in the sacral area. The mass exhibited a more solid cranial component infiltrating the sacrum with cortical breakthrough and a more caudal, complex cystic component extending into the right gluteal soft tissue (Figure 5). Further workup revealed no evidence of distant metastasis.

After a multidisciplinary clinical discussion, the patient has been scheduled for 12 cycles of FOLFIRINOX chemotherapy, followed by completion excision surgery of the sacrum.

## 3. Discussion

Adult SCTs are rare, and malignant transformation within these tumors is exceedingly uncommon, with only a few cases reported in the literature [13, 14].

Adult SCTs most often present as intrapelvic masses, making their detection more challenging compared to the pediatric population, where 90% of cases manifest as visible extrapelvic masses [15].

Although adult SCTs are believed to be present since birth, they may remain undetected for years, increasing the risk of malignant transformation. Delayed medical intervention can result in progression to advanced, unresectable malignancies. Additionally, the rarity of adult SCTs and the lack of a standardized staging system pose significant challenges in selecting the most effective treatment approach.

In the study by Biskup et al., nine cases of teratomas with malignant transformation (TMT) were reported, including two cases of adult SCTs. Of these nine patients, seven underwent surgical resection, with adjuvant chemotherapy administered in one case. Two patients experienced relapse following resection but were subsequently cured with chemotherapy [13].

Wang et al. reported a case of a giant SCT with malignant transformation and lung metastasis in an adult female, highlighting the significance of elevated tumor markers such as CEA, LDH, and AFP in predicting malignant behavior in adult SCTs. However, in our patient, only the CEA level was measured, and it was within the normal range [14].

Several studies have suggested that MRI is the imaging modality of choice for evaluating SCTs, as it effectively assesses the extent of the mass and the involvement of adjacent organs, particularly in cases with a possible malignant component [8, 14, 16].

Considering the rarity of adult SCTs and the even more uncommon occurrence of malignant transformation, it is essential to consider a broad differential diagnosis when evaluating a mass in the sacrococcygeal region of an adult patient. More common entities that should be excluded include metastatic carcinoma, chordoma, and soft tissue sarcomas such as liposarcoma and malignant peripheral nerve sheath tumor. Benign conditions, including pilonidal cysts, rectal duplication cysts, fistulas with presacral extension, and abscesses, should also be considered [16].

Histopathologic examination remains the gold standard for diagnosing SCT. The presence of mature, benign tissues derived from all three germ cell layers supports the diagnosis of SCT. Conversely, features such as infiltrative growth, cytologic atypia, and complex architectural patterns especially—when correlated with the appropriate clinical context—may raise concern for malignant transformation [17, 18].

Immunohistochemistry plays a crucial role in cases of malignant transformation, as malignancies arising from SCTs typically exhibit the immunoprofile of their somatic counterparts. When used alongside appropriate morphology, immunohistochemistry aids pathologists in more accurately subcategorizing the type of malignant transformation. In our case, the presence of a distinct population of duct-like structures and angulated infiltrating glands with eosinophilic cytoplasm and atypical round-to-ovoid nuclei was consistent with pancreatobiliary differentiation. Additionally, loss of SMAD4 nuclear expression provided further support for this diagnosis. The absence of a detectable mass in the pancreatobiliary system served as another helpful clue.

Loss of nuclear SMAD4 staining and loss of p16 nuclear staining are present in approximately 55% and 75% of pancreatic cancers, respectively [19]. In cases with retained nuclear SMAD4 expression, positivity for less specific immunostains such as Cytokeratins 8, 18, and 7, as well as MUC1 and MUC5AC in the context of appropriate morphology, can still be suggestive of pancreatobiliary differentiation [20].

In patients with SCT diagnosed at birth, complete resection is recommended within the first month of life, followed by 6 years of postoperative surveillance to monitor for potential recurrence. Although definitive risk factors for recurrence are not well established, tumor histology and the completeness of resection including coccygectomy have been reported as potential contributing factors [21]. In general, SCTs have an excellent prognosis after complete resection; however, the presence of leptomeningeal involvement or malignant transformation is associated with a poor prognosis [22].

Given that the initial clinical impression in our patient was a pilonidal cyst, which led to incomplete resection of the mass, we recommend that patients with sacrococcygeal masses, particularly those with a persistent mass since childhood, undergo an abdominopelvic MRI followed by complete surgical resection with clear margins. In cases with advanced malignant components, adjuvant chemotherapy may be beneficial. While the optimal chemotherapy regimen depends on the specific nature of the malignant component, the literature currently lacks a standardized approach for treating adult SCTs with malignant transformation.

## 4. Conclusion

SCTs should be excised completely at the time of diagnosis. Although the majority of SCTs are benign, somatic malignant transformation can occur. Delays in seeking treatment may lead to advanced-stage cancer, underscoring the importance of timely and comprehensive management.

## Data Availability

The data that support the findings of this study are available on request from the corresponding author. The data are not publicly available due to privacy or ethical restrictions.
